# NUPR1 is a novel potential biomarker and confers resistance to sorafenib in clear cell renal cell carcinoma by increasing stemness and targeting the PTEN/AKT/mTOR pathway

**DOI:** 10.18632/aging.203012

**Published:** 2021-05-24

**Authors:** Wei He, Fajuan Cheng, Bin Zheng, Jianwei Wang, Guiting Zhao, Zhongshun Yao, Tong Zhang

**Affiliations:** 1 Department of Urology, Shandong Provincial Hospital Affiliated to Shandong First Medical University, Jinan, Shandong, China; 2 Department of Urology, Shandong Provincial Hospital, Cheeloo College of Medicine, Shandong University, Jinan, Shandong, China; 3 Department of Nephrology, Shandong Provincial Hospital Affiliated to Shandong University, Jinan, Shandong, China; 4 Department of Nephrology, Shandong Provincial Hospital, Cheeloo College of Medicine, Shandong University, Jinan, Shandong, China; 5 Department of Urology, Shandong Provincial ENT Hospital Affiliated to Shandong University, Jinan, Shandong, China

**Keywords:** clear cell renal cell carcinoma, NUPR1, therapy resistance, stemness, mTOR

## Abstract

Background: Sorafenib can improve the survival of metastatic clear cell renal cell carcinoma (ccRCC) patients. However, its benefits are modest, as patients eventually become resistant, and the mechanisms remain elusive. NUPR1, a stress-induced protein, has been reported in malignancies and functions as an oncogene by modulating the stress response, facilitating survival in harsh environments and conferring drug resistance. However, its role in ccRCC has not been explored.

Methods: The expression and clinical significance of NUPR1 were analyzed in ccRCC patients in in-house patients and The Cancer Genome Atlas (TCGA) cohorts. The biological functions of NUPR1 were investigated. Xenografts were performed to confirm the effects of NUPR1 on tumorigenesis. The molecular mechanism of NUPR1 was investigated *in vitro* and *in vivo*.

Results: NUPR1 expression was upregulated in tumor tissue. Further analysis showed that NUPR1 overexpression was associated with an aggressive phenotype and predicted a poor prognosis. Depletion of NUPR1 suppressed tumorigenesis and sensitized cells to sorafenib treatment. Finally, mechanistic investigations indicated that NUPR1 promoted tumorigenesis in ccRCC by increasing stemness and activating the PTEN/AKT/mTOR signaling pathway.

Conclusions: Collectively, our results suggest that NUPR1 may serve as a predictor of ccRCC. Notably, NUPR1 silencing reversed sorafenib resistance in ccRCC. These findings provide a novel potential therapeutic target in the clinical management of ccRCC.

## INTRODUCTION

Renal cell carcinomas (RCCs), the most common form of kidney cancers, represent a diverse set of tumors originating from the kidney, including cancers arising from the proximal and distal portions of the nephron, the collecting duct and renal medulla. RCC is the sixth most frequent malignancy in males and the eighth in females, causing more than 15,000 deaths per year in the USA [1]. Although all RCCs receive similar therapeutic regimens, the histologic subtypes are highly heterogeneous in their genetic and molecular alterations, clinical course and therapeutic outcomes [2, 3]. Clear cell renal cell carcinoma (ccRCC) is the most common subtype, accounting for 70%-80% of all cases. ccRCC always contains inactivating mutations of the maternal and paternal copies of the *VHL* (von Hippel-Lindau) gene [4]. Clinically, although most detected tumors are small lesions, one-third of all patients with RCC have metastatic dissemination at the time of diagnosis, and nearly half of all patients die from the disease [5, 6]. Distal metastasis or local recurrence occurs in about 30% of patients after curative surgery of the primary tumor and is associated with a poor prognosis [7]. Systemic therapy targeting the vascular endothelial growth factor (VEGF) and mammalian target of rapamycin (mTOR) offers benefit to metastatic ccRCC. Sorafenib, a commonly used drug, can improve the survival of metastatic ccRCC patients [8]. Unfortunately, ccRCC patients treated with sorafenib eventually become resistant after a median of 6-15 months of treatment [9]. Thus, a better understanding of the mechanisms of resistance to sorafenib is urgently needed.

Nuclear protein 1 (NUPR1), also referred to as p8 or candidate of metastasis (Com-1), is a basic helix-loop-helix chromatin protein [10]. NUPR1 was discovered in pancreatic acinar cells in a study evaluating molecular changes induced by acute pancreatitis in rats [11]. NUPR1 is a stress-induced transcription factor that is abnormally expressed in a wide spectrum of malignancies [12–14]. NUPR1 has recently elicited great attention for its role in several protumorigenic processes, including cell cycle regulation, matrix remodeling, autophagy, apoptosis, senescence and the DNA repair response [15–20]. Notably, NUPR1 is also involved in resistance to antitumor drugs [12, 21, 22]. Therefore, NUPR1 is a promising therapeutic target for developing new cancer therapies. However, the role and prognostic value of NUPR1 in ccRCC remain to be fully elucidated.

The aims of this study were to comprehensively analyze the prognostic value and possible mechanism of NUPR1 in ccRCC. The results suggested that NUPR1 may predict the outcome of ccRCC. NUPR1 dysregulation also promoted ccRCC progression and sorafenib resistance. Mechanistic studies showed that this process was mediated by increasing stemness and the PTEN/AKT/mTOR pathway. NUPR1 merits further investigation as a potential diagnostic marker and therapeutic strategy for ccRCC patients.

## RESULTS

### NUPR1 expression is upregulated and predicts a poor prognosis in ccRCC patients

**Figure 1:**
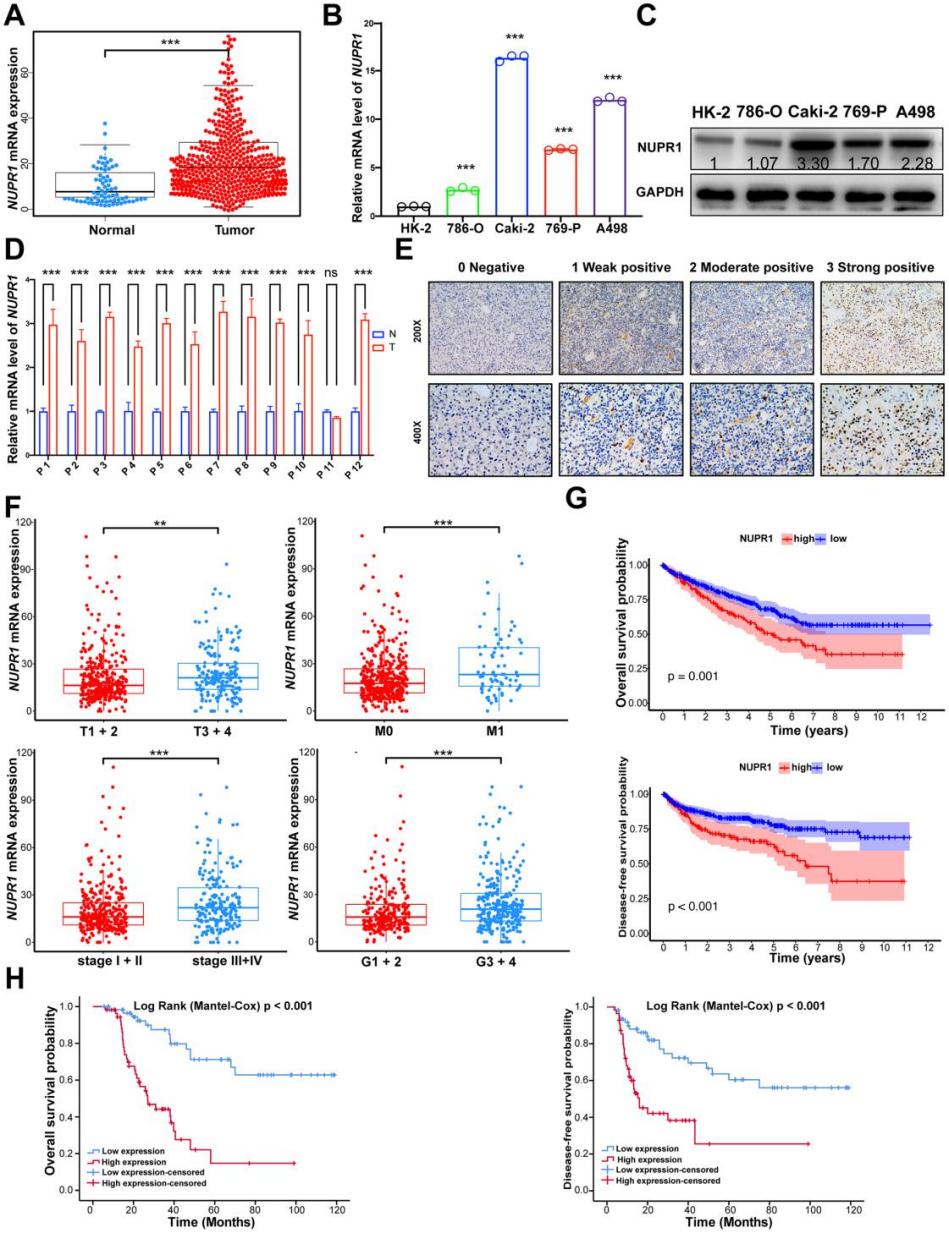
**NUPR1 upregulated is predominantly found in ccRCC and associated with poor prognosis.** (**A**) Comparison of NUPR1 mRNA expression between ccRCC and normal kidney tissue from TCGA-KIRC dataset. (**B**) Relative mRNA level of NUPR1 in ccRCC cell lines compared with human renal proximal tubular epithelial cell line HK-2. (**C**) Western blot showed NUPR1 protein expression in ccRCC cell lines and HK-2. (**D**) Relative mRNA level of NUPR1 in twelve ccRCC tissues and paired normal kidney tissues. (**E**) Immunostaining of NUPR1 expression in 117 ccRCC tissues. Immunostaining intensity was scored as 0 negative, 1 weak positive, 2 moderate positive and 3 strong positive. (**F**) Overexpression of NUPR1 was associated with higher pathologic T stage, metastasis, elevated clinical stage and histologic grade in TCGA-KIRC dataset. (**G**) Kaplan-Meier curves of overall survival and disease-free survival time between low (n=135) and high (n=389) NUPR1 mRNA level groups in TCGA-KIRC dataset. The cutoff value was the mean of NUPR1 mRNA level in 524 ccRCC tissues. (**H**) Kaplan-Meier curves of overall survival and disease-free survival time between low (n=59) and high (n=58) NUPR1 immunostaining intensity groups in cohorts from Shandong Provincial Hospital. The median IHC score was used as the cutoff value. (*p < 0.05, **p < 0.01, ***p < 0.001). (N.S., No statistical significance). ccRCC: clear cell renal cell carcinoma; KIRC: Kidney renal clear cell carcinoma; TCGA: The Cancer Genome Atlas database.

To preliminarily investigate the transcription profile of NUPR1 in RCC, we first analyzed the RNA-seq dataset derived from The Cancer Genome Atlas (TCGA) patients with clear cell RCC (KIRC), chromophobe RCC (KICH) and papillary RCC (KIRP). The data showed that *NUPR1* mRNA expression was increased significantly in ccRCC tissues, KICH and KIRP, compared to adjacent normal kidney tissues (Figure 1A and Supplementary Figure 1A, 1B).

Next, we performed qRT-PCR in ccRCC cell lines and 12 paired human ccRCC tissues. *NUPR1* mRNA levels were significantly increased in ccRCC cells and cancer tissues compared with HK-2 cells (human renal cortex/proximal tubular epithelial cells) and adjacent normal kidney tissues (Figure 1B, 1D). Accordingly, western blot assays showed similar results, with NUPR1 protein expression being upregulated in ccRCC cell lines (Figure 1C).

To assess the correlation between the *NUPR1* transcription level and clinicopathological characteristics, we assessed the TCGA-KIRC data. As shown in Figure 1F, the group with high *NUPR1* mRNA expression was strongly correlated with an elevated pathologic T stage, metastasis, clinical stage and nuclear grade. Subsequently, 117 samples from Shandong Provincial Hospital were analyzed by immunohistochemical staining to further explore the protein expression of NUPR1 (Figure 1E). NUPR1 expression gradually increased along with an increase in pathologic T stage, lymph node involvement, metastasis and stage (Table 1). Collectively, these results indicated that elevated NUPR1 was significantly associated with advanced clinicopathological features in ccRCC.

**Table 1:** Correlation between NUPR1 immunostaining intensity and clinicopathological features in 117 ccRCC patients.

**Clinicopathological features**		**NUPR1 expression**		**p**
		**Low (n= 59)**	**High (n= 58)**	
Gender	Female	19	17	0.842
	Male	40	41	
Age (years)	≤ 65	27	24	0.71
	>65	32	34	
Laterality	Left	36	27	0.14
	Right	23	31	
Pathological T	T1+2	38	25	0.026*
	T3+4	21	33	
Pathological N	N0	56	42	0.001**
	N1	3	19	
Pathological M	M0	59	46	<0.001***
	M1	0	12	
Histologic grade	G1+2	39	41	0.692
	G3+4	20	17	
Stage	I+II	39	25	0.016 *
	III+IV	20	33	

ccRCC: clear cell renal cell carcinoma (*p < 0.05, **p < 0.01, ***p < 0.001).

To explore the prognostic significance of NUPR1, we analyzed the TCGA-KIRC dataset using a Kaplan-Meier analysis with a log-rank test. As shown in Figure 1G, the high NUPR1 mRNA level in primary tumors was correlated with poor overall survival (OS) and disease-free survival (DFS). Similarly, the immunostaining results demonstrated that patients with elevated NUPR1 protein expression experienced shorter OS and DFS (Figure 1H). Univariate and multivariate Cox regression analyses revealed that higher pathologic T stage, metastasis and high immunostaining intensity of NUPR1 were unfavorable prognostic factors in ccRCC patients, (OS, HR = 2.263, 95% CI = 1.061– 4.827, *p* = 0.035, Table 2; DFS, HR = 2.031, 95% CI = 1.021– 4.040, *p* = 0.043, Table 3). Thus, we hypothesized that NUPR1 might play important roles in the progression of ccRCC.

**Table 2:** Uni- and multi-variate Cox regression of NUPR1 protein expression for overall survival in 117 ccRCC.

**Variables**	**Univariate analysis**				**Multivariate analysis**		
	**HR**	**95% CI**	**p**		**HR**	**95% CI**	**p**
NUPR1 expression	4.862	2.526 - 9.357	<0.001***		2.263	1.061 - 4.827	0.035*
High Vs. Low							
Gender	1.502	0.762 - 2.958	0.240				
Male Vs. Female							
Age (years)	1.140	0.633 - 2.054	0.661				
>65 ≤ 65							
Laterality	1.160	0.650 - 2.069	0.616				
Right Vs. Left							
Pathological T	7.437	3.654 - 15.135	<0.001***		6.799	1.757 - 26.313	0.006**
T3+4 Vs. T1+2							
Pathological N	3.090	1.488 - 6.414	0.002**		1.604	0.671 - 3.838	0.288
N0 Vs. N1							
Pathological M	8.433	4.028 - 17.652	<0.001***		3.838	1.561 - 9.441	0.003**
M1 Vs. M0							
Histologic grade	1.559	0.860 - 2.826	0.144				
G3+4 Vs. G1+2							
Stage	5.627	2.913 - 10.870	<0.001***		0.575	0.157 - 4.827	0.402
III+IV Vs. I+II							

CI: confidence interval; ccRCC: clear cell renal cell carcinoma; HR: hazard ratio. (*p < 0.05, **p < 0.01, ***p < 0.001).

**Table 3:** Uni- and multi-variate Cox regression of NUPR1 protein expression for disease-free survival in 117 ccRCC.

**Variables**	**Univariate analysis**				**Multivariate analysis**		
	**HR**	**95% CI**	**p**		**HR**	**95% CI**	**p**
NUPR1 expression	3.264	1.772 - 6.012	<0.001***		2.031	1.021 - 4.040	0.043*
High Vs. Low							
Gender	1.066	0.580 - 1.957	0.837				
Male Vs. Female							
Age (years)	0.979	0.557 - 1.720	0.940				
>65 ≤ 65							
Laterality	1.358	0.774 - 2.382	0.286				
Right Vs. Left							
Pathological T	5.782	2.937 - 11.383	<0.001***		5.319	1.271 - 22.253	0.022*
T3+4 Vs. T1+2							
Pathological N	2.465	1.203 - 5.053	0.014*		0.989	0.450 - 2.174	0.978
N0 Vs. N1							
Pathological M	16.800	7.606 - 37.110	<0.001***		6.820	2.893 - 16.078	<0.001***
M1 Vs. M0							
Histologic grade	1.312	0.726 - 2.369	0.368				
G3+4 Vs. G1+2							
Stage	4.872	2.554 - 9.293	<0.001***		0.720	0.178 - 2.918	0.646
III+IV Vs. I+II							

CI: confidence interval; ccRCC: clear cell renal cell carcinoma; HR: hazard ratio. (*p < 0.05, **p < 0.01, ***p < 0.001).

### NUPR1 promotes ccRCC proliferation *in vitro*

**Figure 2:**
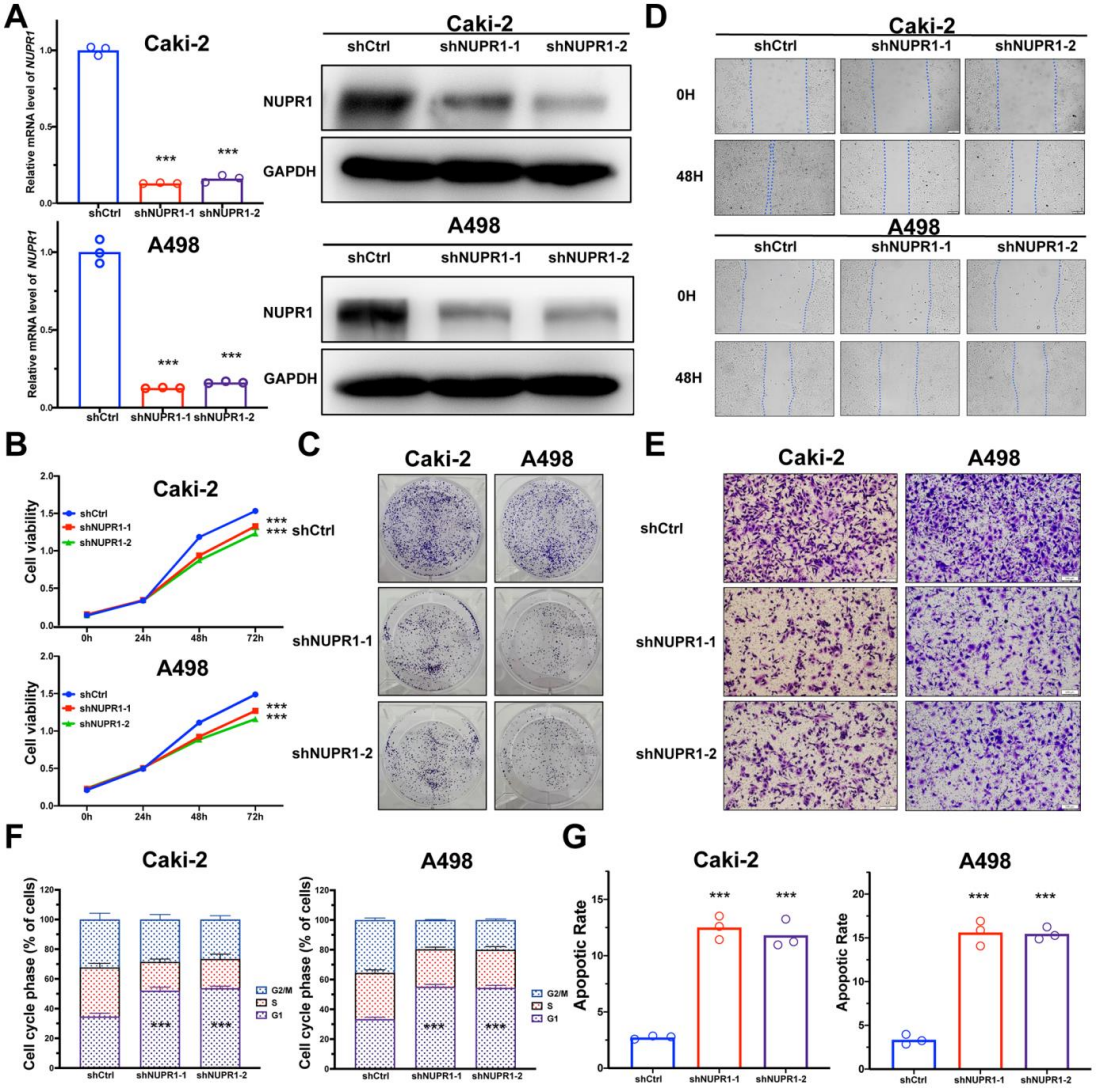
**NUPR1 facilitated tumorigenesis of ccRCC *in vitro*.** (**A**) Verification of NUPR1 mRNA and protein knockdown in ccRCC cell lines. (**B**) Cell growth curves of CCK-8 assay for ccRCC cell lines with NUPR1 silencing. (**C**) Colony formation assay for ccRCC cells with NUPR1 depletion. (**D**) The wound-healing assay of NUPR1 silencing on the migration of ccRCC cells. (**E**) Transwell experiment analysis of the effect of NUPR1 depletion on migratory and invasive abilities of ccRCC cells. (**F**) Effects of NUPR1 silencing on cell cycle regulation using flow cytometry. (**G**) Apoptosis assay of silencing NUPR1 in ccRCC by flow cytometry. (*p < 0.05, **p < 0.01, ***p < 0.001). CCK-8: cell counting kit-8; ccRCC: clear cell renal cell carcinoma.

To identify the pathological function of NUPR1 in ccRCC, we synthesized two shRNAs specifically targeting NUPR1. We found that the shRNAs remarkably inhibited the expression of NUPR1 in Caki-2 and A498 cells (Figure 2A). Subsequently, we analyzed the effect of NUPR1 on ccRCC cell proliferation by conducting CCK-8 and colony formation assays. The results showed that knockdown of NUPR1 repressed the growth of ccRCC cells (Figure 2B). Moreover, colony formation assays showed that the colony numbers were significantly reduced after NUPR1 depletion (Figure 2C and Supplementary Figure 2A).

### NUPR1 enhances the aggressive abilities of ccRCC cells *in vitro*

To further investigate the role of NUPR1 to promote ccRCC metastasis, we analyzed the migratory and invasive abilities of ccRCC cells by manipulating NUPR1 expression. To inhibit the expression of NUPR1 in ccRCC cells, two shRNAs were transduced into Caki-2 and A498 cells. The wound healing assay indicated that NUPR1 knockdown predominantly suppressed metastatic function in both Caki-2 and A498 cells (Figure 2D and Supplementary Figure 2B). Further, the depletion of NUPR1 significantly reduced the migratory and invasive abilities of Caki-2 and A498 cells in the Transwell assay (Figure 2E and Supplementary Figure 2C).

### NUPR1 knockdown induces G_0_/G_1_ arrest and promotes ccRCC cell apoptosis

To elucidate the mechanism underlying NUPR1 promotion of cell growth and proliferation, cell cycle and cell apoptosis regulation were assessed via flow cytometry. As shown in Figure 2F, decreased expression of NUPR1 notably reduced the proportion of cells in the S phase and G_2_/M phases of mitosis; in contrast, more cells were arrested in the G_0_/G_1_ phases (Supplementary Figure 3A). The FACS analysis illustrated that NUPR1 deficiency efficaciously facilitated the apoptosis of Caki-2 and A498 cells (Figure 2G and Supplementary Figure 3B). These results showed that NUPR1 depletion inhibited cell cycle progression and induced cell apoptosis.

### NUPR1 knockdown inhibits the growth of ccRCC cells *in vivo*

**Figure 3:**
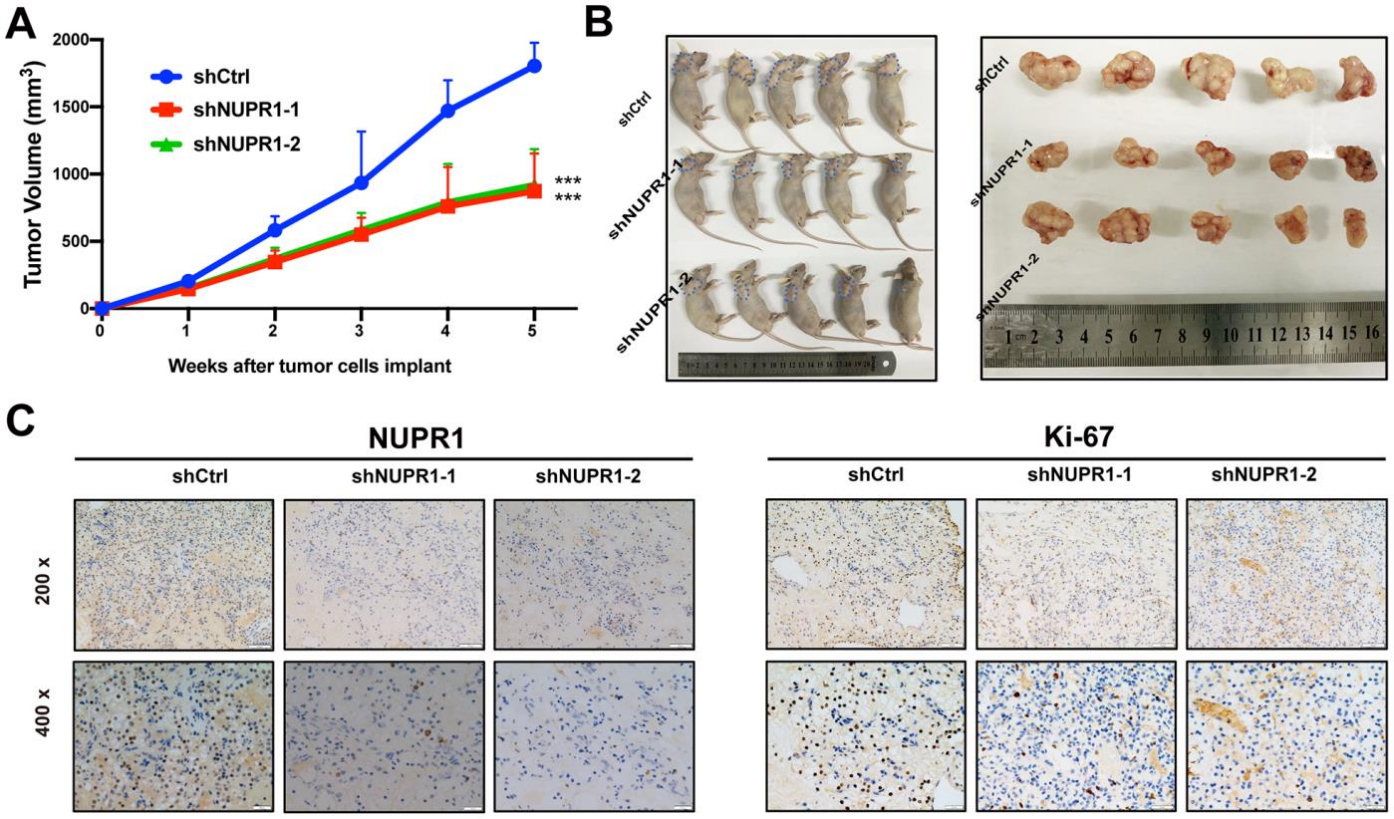
**NUPR1 promoted aggressive abilities of ccRCC *in vivo*.** (**A**) Growth curves of subcutaneous xenografts in nude mice (n=5). (**B**) Photographs of nude mice and xenografts. (**C**) NUPR1 and Ki-67 expression were detected by IHC in xenografts sections. (*p < 0.05, **p < 0.01, ***p < 0.001). ccRCC: clear cell renal cell carcinoma; IHC: Immunohistochemistry.

To assess the effect of NUPR1 on ccRCC tumorigenicity *in vivo*, we established a xenograft mouse model in which Caki-2 cells transfected with shCtrl, shNUPR1-1 or shNUPR1-2 were inoculated subcutaneously into the dorsal regions of nude mice. The xenograft assay demonstrated that the xenograft tumors in mice that received NUPR1-depleted cells were significantly reduced in terms of weight and volume compared with the control group (Figure 3A, 3B). Moreover, immunohistochemical staining revealed that Ki-67 expression was remarkably decreased in NUPR1-depleted xenografts, indicating that the knockdown of NUPR1 attenuated tumor proliferation (Figure 3C).

### Downregulation of NUPR1 increases sensitivity to sorafenib in ccRCC

**Figure 4:**
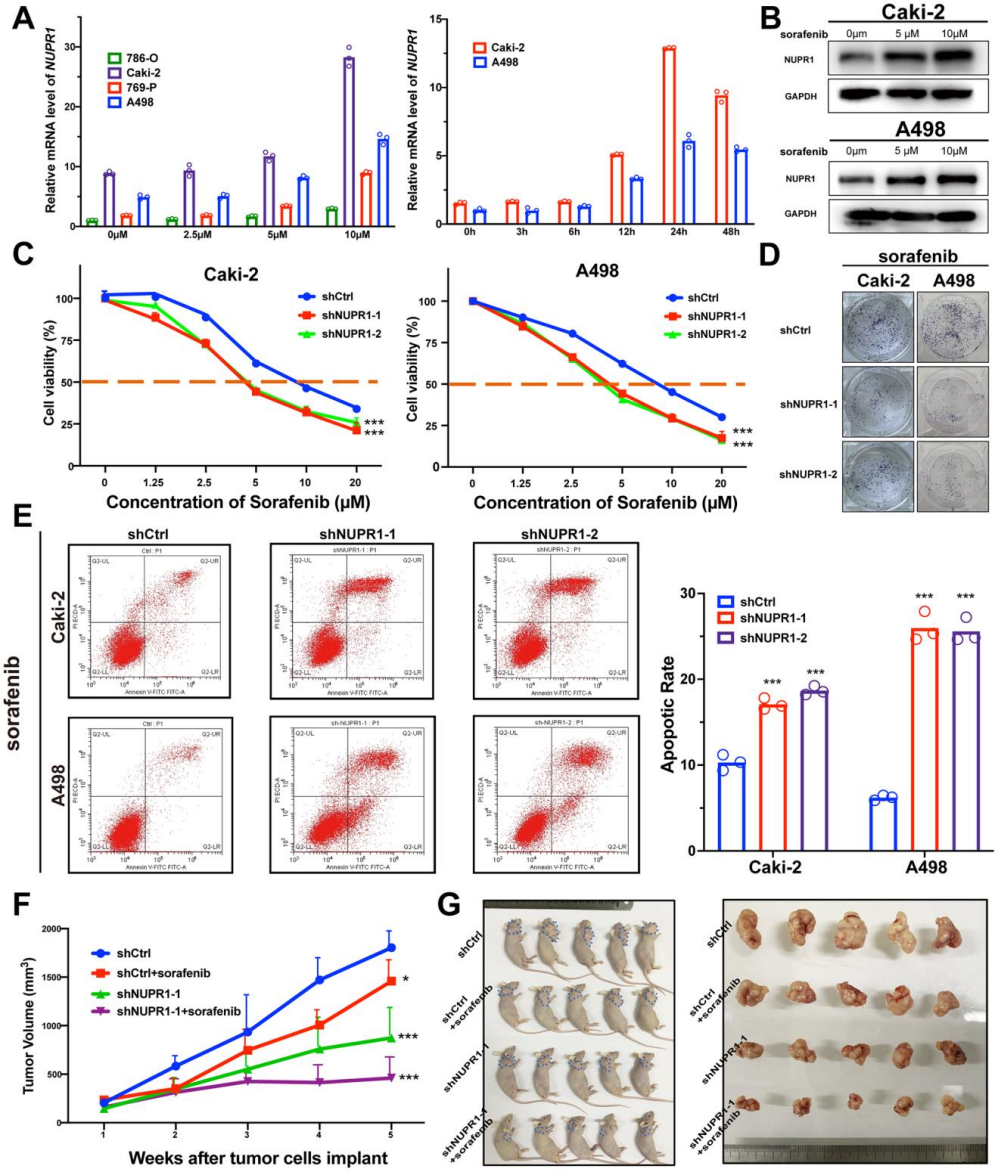
**Depletion of NUPR1 promoted sensitivity to sorafenib in ccRCC.** (**A**) Time and concentration-dependent manner of sorafenib treatment on NUPR1 expression in ccRCC cell lines verified by qRT-PCR. (**B**) Western blot assay of sorafenib inducing NUPR1 protein expression. (**C**) CCK-8 assay of NUPR1 silencing after sorafenib treatment at the indicated concentrations for 24h. The IC50 values were 10.26, 5.28, 5.86 μM for shCtrl, shNUPR1-1, shNUPR1-2, respectively in Caki-2. The IC50 values were 8.73, 4.55, 4.36 μM, respectively in A498. (**D**) Colony formation experiments of NUPR1 silencing after sorafenib (5μM) treatment for 2 weeks. (**E**) Flow cytometry analysis of effects of NUPR1 depletion on apoptosis after sorafenib (5μM) treatment for 24h. (**F**) Growth curves of subcutaneous xenografts in nude mice (n=5) under different treatments. (**G**) Images of nude mice and anatomical picture of subcutaneous xenografts. (*p < 0.05, **p < 0.01, ***p < 0.001). CCK-8: cell counting kit-8; ccRCC: clear cell renal cell carcinoma; IC50: 50% inhibiting concentration; qRT-PCR: quantitative real-time reverse transcription PCR.

ccRCC is a lethal urologic malignancy that causes the most deaths, most of which are a result of relapse or resistance to tyrosine kinase inhibitor (TKI) treatment, usually sorafenib. Therefore, we thoroughly explored whether the suppression of NUPR1 expression in ccRCC increases sensitivity to sorafenib treatment. As shown in Figure 4A, 4B, NUPR1 expression was increased after sorafenib treatment. *NUPR1* mRNA transcription was induced in ccRCC cell lines in a dose- and time- dependent manner. Consistent with previous results, immunoblotting showed that exposure to sorafenib promoted NUPR1 protein expression (Figure 4B). Then, we cultured ccRCC cells in different concentrations of sorafenib to calculate the IC50 *in vitro*. The results indicated that Caki-2 and A498 cells expressing shNUPR1-1 or shNUPR1-2 were more sensitive, with a lower IC50 for sorafenib than control cells (Figure 4C) and a decreased capacity for cell proliferation and colony formation (Figure 4D and Supplementary Figure 4A). Consistently, FACS demonstrated that exposure to sorafenib led to an increased apoptotic rate among NUPR1 depleted cells (Figure 4E and Supplementary Figure 4B). However, the sorafenib does not induce cell cycle arrest (Supplementary Figure 4C, 4D). Together, these data indicated that NUPR1 was required for sorafenib resistance *in vitro*.

To validate these data *in vivo*, we utilized xenografts established with Caki-2 cells expressing shCtrl or shNUPR1. Each group was treated with either sorafenib (40 mg/kg per day) or saline solution once the tumor reached a diameter of 5 mm. As shown in Figure 4F, 4G, sorafenib treatment caused a reduction in tumor volume compared to that of control xenografts. The intensity of ki-67 in xenograft with NUPR1 depletion and sorafenib treatment decreased more significantly compared to NUPR1 depletion or sorafenib treatment alone (Supplementary Figure 4E). Furthermore, there also was synergism of NUPR1 silencing and sorafenib in promoting apoptosis in xenografts in TUNEL assay (Supplementary Figure 4F).

Taken together, these data demonstrated that NUPR1 depletion increased sensitivity to sorafenib both *in vitro* and *in vivo*.

### Reduced expression of *NUPR1* leads to deceased stemness in ccRCC cells

**Figure 5:**
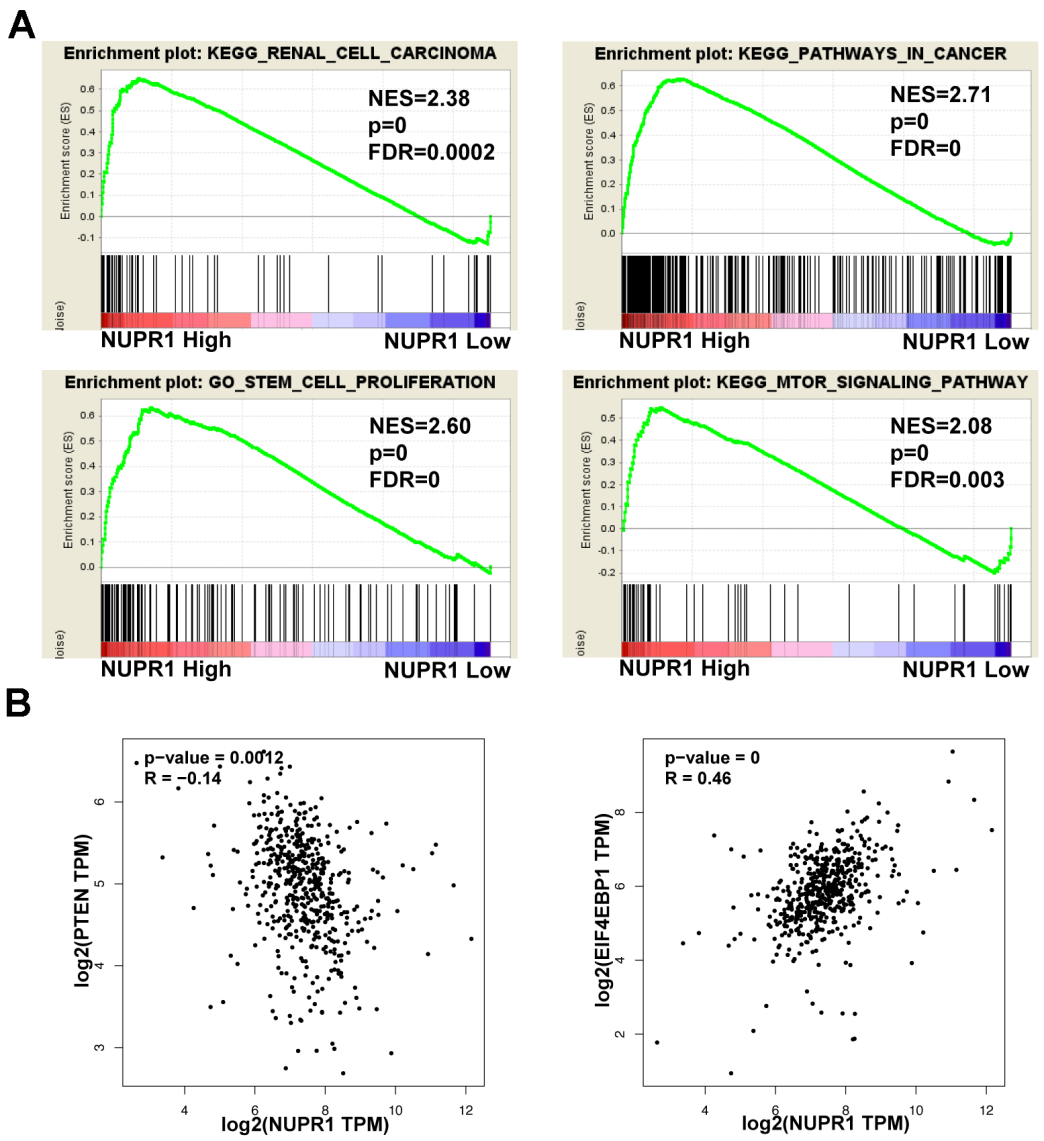
**Pathways involved in the pathogenesis of NUPR1 with GSEA and correlation analysis between NUPR1 and PTEN, 4EBP1 in TCGA-KIRC cohort.** (**A**) Enrichment curves for activated gene sets using GSEA pathway analysis. (**B**) Correlation between NUPR1 and 4EBP1 in TCGA-KIRC dataset. (*p < 0.05, **p < 0.01, ***p < 0.001). GSEA: gene set enrichment analysis; KIRC: Kidney renal clear cell carcinoma; TCGA: The Cancer Genome Atlas database.

To further explore the underlying mechanisms by which NUPR1 promoted ccRCC tumorigenesis, we performed GSEA based on TCGA data form and identified several enriched pathways. Notably, enrichment analysis showed that overexpression of NUPR1 was associated with the cancer pathway, cancer cell stemness, mTOR pathway and renal cell carcinoma (Figure 5A).

**Figure 6:**
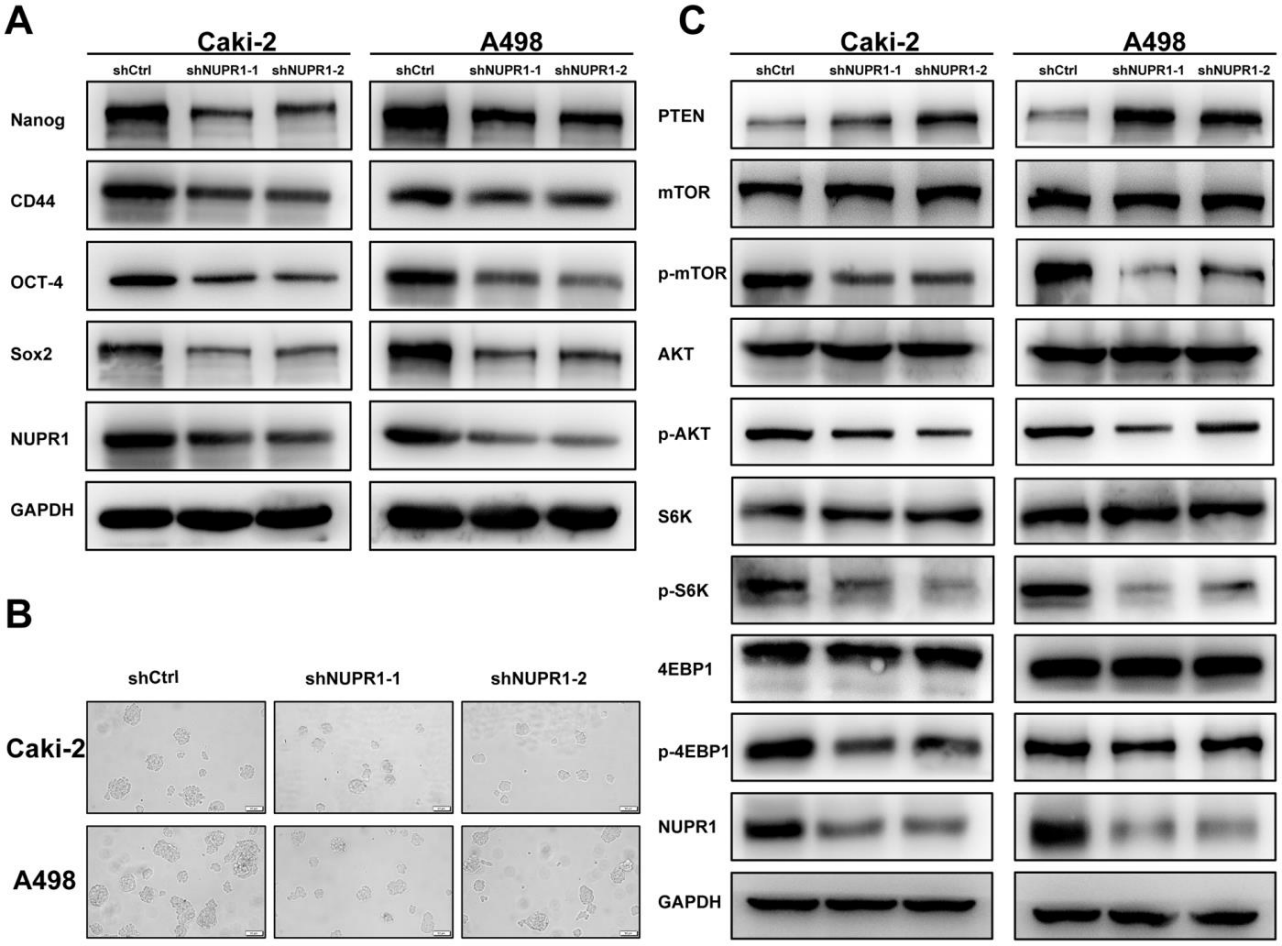
**NUPR1 silencing mediated stem-like properties and suppressed the PTEN/AKT/mTOR signaling pathway in ccRCC.** (**A**) Western blot analysis of effects of NUPR1 depletion on stemness-related biomarkers. (**B**) Tumor sphere assay was used for analysis of cancer stemness for NUPR1 silencing. (**C**) knockdown of NUPR1 increased PTEN and decreased the protein levels of p-mTOR, p-AKT, p-S6K and p-4EBP1 in ccRCC cells. (*p < 0.05, **p < 0.01, ***p < 0.001).

To investigate the relevance of NUPR1 to the stem-like phenotype, stem cell biomarkers were assessed using qRT-PCR and western blot. We demonstrated that the mRNA and protein levels of Nanog, CD44, OCT-4 and Sox2 were significantly decreased in ccRCC cells transfected with shNUPR1 compared with control cells (Figure 6A and Supplementary Figure 5A, 5B). Moreover, the sphere assay revealed that a notably smaller size of spheres formed in culture and that a smaller number of spheres was also observed when NUPR1 was inhibited (Figure 6B). Collectively, these data suggested that NUPR1 played a role in the transition to a stem-like phenotype in ccRCC cells.

### NUPR1 depletion suppresses ccRCC by activating the PTEN/AKT/mTOR pathway in ccRCC cells

As the PTEN/AKT/mTOR pathway regulates stemness and metastasis and it is frequently activated in ccRCC, we examined the effect of NUPR1 on this signaling pathway. We conducted a correlation analysis between NUPR1 and the PTEN and 4EBP1 in GEPIA database (http://gepia.cancer-pku.cn) (Figure 5B). As shown in Figure 6C, a decrease in key proteins in the pathway, specifically phosphorylated AKT, mTOR, S6K and 4EBP1 but not in the total amount of these molecules, was observed in Caki-2 and A498 cells with NUPR1 knockdown compared with control cells. Moreover, PTEN, the negative regulator of the AKT/mTOR signaling pathway, was increased in NURP1-knockdown cells.

## DISCUSSION

Renal cell carcinoma, one of the most common urological malignancies, is characterized by frequent inactivation of the *VHL* gene and hyperactivation of the HIF-VEGF axis, leading to abundant vascularization within tumors. Over one-third of ccRCC patients harbor metastasis with a very low five-year survival rate [23]. Although TKI drugs such as sunitinib, sorafenib and axitinib greatly benefit metastatic ccRCC patients, they do not produce a durable response, as ccRCC patients inevitably acquire drug resistance [24]. Thus, there is an urgent need to elucidate the molecular mechanism(s) of resistance to TKI therapy.

Cancer cells can develop mechanisms to adapt to stressful environmental conditions, such as hypoxia, deprivation of nutrition and drugs [25–28]. These adaptations activate several stress proteins to facilitate cancer cell survival, growth, development and progression. Recently, accumulating evidence has revealed that cancer cell biological function relies highly on these stress-induced proteins [29, 30]. Thus, understanding the mechanisms of stress factors could shed light on drug resistance and mine promising therapeutic targets in cancer treatment.

NUPR1 is a stress-response transcription factor upregulated by many biological and chemical stressors [31]. It was first reported to be activated in the acute phase of pancreatitis [11]. NUPR1 has been demonstrated to participate in many malignancy-related processes, including regulation of the cell cycle [32], apoptosis [33], senescence [17], DNA damage response [34], metastasis [19] and autophagy [35]. NUPR1 dysregulation has been reported in several malignancies, including breast cancer [36], pancreatic cancer [11, 37], lung cancer [35], prostate cancer [38], colorectal cancer [39] and glioma [40]. Several studies have shown that NUPR1 plays a key role in antidrug resistance by mediating autophagy and antiapoptotic activities [41, 42]. However, the role and prognostic value of NUPR1 in ccRCC remain unexplored.

According to TCGA dataset, overexpression of NUPR1 was observed in a variety of solid tumors including three RCC subtypes (KIRC, KICH, KIRP), glioblastoma multiforme (GBM) and lymphoma, compared to that in the corresponding normal tissues (Supplementary Figure 1A). In this study, we assessed NUPR1 expression in cancer cell lines, clinical ccRCC samples and adjacent kidney tissue. The results revealed that NUPR1 was overexpressed in ccRCC tissues. Furthermore, NUPR1 upregulation was associated with elevated pathologic T stage, clinical stage and nuclear grade (Figure 1F). Moreover, high NUPR1 expression promoted metastasis, suggesting that NUPR1 is a promising biomarker for predicting progression. ccRCC patients expressing higher NUPR1 exhibited a lower OS and DFS probability than those with lower NUPR1 levels (Figure 1G, 1H). Thus, we propose NUPR1 as a prognostic candidate for individual stratification of ccRCC subgroups, which might benefit from more personalized medicine. In addition, we also observed that silencing NUPR1 resulted in decreased cell proliferation, colony formation, migration and invasion (Figure 2 and Supplementary Figure 2).

Sorafenib has been shown to effectively inhibit vascularization and suppress tumor progression [43]. However, the majority of advanced ccRCC patients who receive sorafenib exhibit progression within 15 months of treatment [9]. The mechanisms of sorafenib resistance remain complex and unclarified. Anticancer drug resistance has always been a major challenge for ccRCC treatment. Therefore, there is an intense focus on studies of the drug resistance.

Several studies have demonstrated that NUPR1 transcriptionally regulates the expression of several drug resistance-associated genes to mediate drug resistance in various malignancies [22, 38, 44]. A previous study reported that NUPR1 mediated sorafenib resistance in hepatocellular carcinoma [42]. However, the role of NUPR1 in sorafenib resistance has not been elucidated in ccRCC. Our results revealed that the expression of NUPR1 could be induced by sorafenib and that this upregulation mediated the resistance of ccRCC cells to sorafenib. Furthermore, downregulation of NUPR1 by shRNA promoted the sensitivity of ccRCC cells to sorafenib both *in vitro* and *in vivo*. These findings render NUPR1 a promising target for the treatment of sorafenib-resistant ccRCC. The mechanism of NUPR1 silencing in reducing resistance to sorafenib may be multifactorial.

Cancer stem cells, a small subgroup of tumor cells, have self-renewal and multipotency and play an important role in carcinogenesis, progression and drug resistance [45]. Recently, several studies reported that sorafenib resistance is associated with activated stemness of cancer cells in hepatocellular carcinoma [46, 47]. Thus, inhibition of cancer cell stemness is a potential strategy for of reversing drug resistance. Emma and his colleagues reported that NUPR1 was involved in sorafenib resistance and that silencing of NUPR1 inhibited cell growth migration and increased sensitivity to sorafenib [42]. However, there is no evidence to prove that NUPR1 regulates the cancer stemness and sorafenib resistance in ccRCC. Here, our data showed that sorafenib upregulated NUPR1 expression. Furthermore, we demonstrated that NUPR1 depletion led to a significant reversal of the resistance to sorafenib in ccRCC, which involved the downregulation of stemness-associated genes, including Nanog, CD44, Sox2 and Oct-4.

The PTEN/AKT/mTOR axis plays a pivotal role in cell growth, invasion, metastasis and drug resistance in ccRCC [48, 49]. AKT/mTOR signaling is frequently activated in ccRCC and is associated with progression and poor survival [23]. Therefore, new therapeutic strategies targeting this pathway may overcome drug resistance and improve clinical outcomes. By GSEA analysis, we discovered that AKT/mTOR signaling was positively correlated with NUPR1 (Figure 5A), suggesting that NUPR1 might promote sorafenib resistance by AKT/mTOR signaling. Here, we showed that depletion of NUPR1 promoted PTEN expression and suppressed AKT/mTOR signaling in ccRCC cells (Figure 6C). Mechanistic investigation revealed that AKT/mTOR pathway activation was important for the oncogenic properties of NUPR1 in ccRCC.

## CONCLUSIONS

To date, this is the first work to investigate the role of NUPR1 in ccRCC. We have demonstrated that overexpression of NUPR1 is closely associated with metastatic features and a worse prognosis in ccRCC patients. Downregulation of NUPR1 can decrease ccRCC cell growth and metastasis *in vitro* and *in vivo*. Notably, NUPR1 facilitates the proliferation and migration of ccRCC cells by promoting stemness and activating the PTEN/AKT/mTOR signaling pathway. Here, we report that NUPR1 silencing reverses sorafenib resistance in ccRCC. Collectively, our data suggest that NUPR1 serves as a promising prognostic biomarker in ccRCC and functions as an oncogene to promote tumorigenesis. Targeting NUPR1 may represent a novel potential therapeutic strategy in the clinical management of ccRCC.

## MATERIALS AND METHODS

### In silico analysis

NUPR1 expression levels in RCC (KIRC, KICH, KIRP) specimens and the correlated clinical data, including TNM stage, tumor grade, overall survival (OS), and disease-free survival (DFS), were downloaded from The Cancer Genome Atlas database (TCGA; https://xenabrowser.net/heatmap/). Gene set enrichment analysis (GSEA; http://software.broadinstitute.org/gsea/index.jsp) was performed to determine signaling pathways and molecules involved in the pathogenesis of ccRCC when NUPR1 was highly expressed. The NUPR1 mean level was used as the cutoff criterion.

### Patients, tissue specimens and follow-up

A total of 117 patients with primary ccRCC who underwent surgery between January 2010 and December 2019 at Shandong Provincial Hospital were included in this study. No patients had received targeted therapy, chemotherapy or radiotherapy prior to surgery. The entire procedure was approved by the Ethics Review Committee of the Shandong Provincial Hospital. The study was performed in accordance with the Declaration of Helsinki and the guidelines of the committee. Written informed consent was obtained from each patient in the study. The paraffin-embedded tissue of the patient in the study was re-embedded into new blocks for Immunohistochemical staining. Pathological specimens and clinicopathological characteristics were collected, and all samples were anonymous. Patients were followed up from the date of surgery, with a mean follow-up of 39 months. Overall survival was defined as the interval between surgery and death from any cause or the last follow-up. Disease-free survival was calculated as the interval between the initial surgery and disease progression or censoring at the time of last follow-up.

### Immunohistochemical (IHC) staining

Slides were stained with antibodies against NUPR1(ab234696, Abcam, Shanghai), and Ki-67(ZM-0166, ZSGB-Bio, Beijing), according to standard immunoperoxidase-staining procedures. Positive staining for NUPR1 and Ki-67 was observed in the nuclei [50]. The sections were evaluated by two independent pathologists who calculated their corresponding IHC score. Five fields of vision were randomly selected per section at a magnification of 400 ×. The scores were recorded as four grades (0-3) based on the quantity of immunoreactive cells. The semiquantitative analysis of NUPR1 staining using a 4-grade scale was defined as follows: sections with no labeling or labeled cells < 5% were scored as 0, sections with 5–25% of labeled cells were scored as 1, with 25–50% of labeled cells as 2, and with >50% of labeled cells as 3. The median IHC score was used as the cutoff value to separate patients into high and low NUPR1 expression groups.

### Cell culture

HEK293T cells, the human ccRCC cell lines 786-O, 769-P, A498, and Caki-2 and the human renal cortex/proximal tubular epithelial cell line HK-2 were obtained from the Type Culture Collection Cell Bank, Chinese Academy of Science Committee (Shanghai, China). HEK293T cells were grown in DMEM (Gibco, USA). 786-O and 769-P cells were grown in RPMI 1640(Gibco, USA). A498 were grown in McCoy’s 5A (Gibco, USA). HK-2 cells were grown in DMEM/Hams F12 (Gibco, USA). All media were supplemented with 10% fetal bovine serum (Gibco, USA), 100 U/mL penicillin and 100 ug/mL streptomycin (Invitrogen). Cells were maintained in a humidified incubator at 37° C and 5% CO_2_.

### Lentivirus shRNA-mediated knockdown of NUPR1

Two shRNAs (shRNA1:5’-CCGGGGATGAATCTGACCTCTATAGCTCGAGCT-ATAGAGGTCAGATTCATCCTTTTTG-3’; shRNA2:5’-CCGGGAGAGGAAACT-GGTGACCAAGCTCGAGCTTGGTCACCAGTTTCCTCTCTTTTTG-3’) were constructed to target NUPR1 in subsequent experiments. Nontarget shRNA (sequence: 5’-CCGGGCGCGATAGCGCTAATAATTTCTCGAGAAATTATTAGC GCT ATCGCGCTTTT-3’) was also constructed. shNUPR1 and nontarget shRNA were inserted into a GenePharma supersilencing vector (pGLVH1/GFP-puromycin). Recombinant lentiviruses expressing NUPR1 shRNA or nontarget shRNA (shNUPR1 and shCtrl, respectively) were produced by GenePharma (Shanghai, China). Cells were transduced with concentrated virus, and stable clones were selected with puromycin (Sigma) for two weeks. Knockdown of NUPR1 expression at the mRNA level was confirmed by qRT-PCR as discussed below.

### RNA extraction and quantitative real-time reverse transcription PCR (qRT-PCR)

Total RNA of frozen tissue or cell lines was isolated and purified using Invitrogen TRIzol Reagent (Thermo Fisher Scientific) following the manufacturer’s protocol. The mRNA was reverse transcribed into cDNA using a standard procedure with a TaqMan Reverse Transcriptase Kit (Qiagen, Valencia, CA). SYBR green-based quantitative real-time PCR was subsequently carried out with a 7500 ABI detection system. Relative expression was determined by the 2^−ΔΔCt^ method. The primer sequences used are listed in Supplementary Table 1.

### SDS-PAGE and western blotting

Cells were lysed using RIPA buffer (R0010, Solarbio, Beijing), and total protein was extracted. After concentration was determined, aliquots of 100 μg of total protein and Tricolor Prestained Protein Marker (PR1930, Solarbio, Beijing) were mixed with buffer and loaded into each well of an SDS-PAGE gel for subsequent electrophoresis and then transferred to PVDF membranes which were blocked with 5% nonfat milk. The samples were then incubated with primary antibodies against GAPDH (ab8245, Abcam, Shanghai), NUPR1 (ab6028, Abcam, Shanghai), PTEN (9552S, Cell Signaling Technology), mTOR (2972, Cell Signaling Technology), p-mTOR (2971, Cell Signaling Technology), AKT (8805, Cell Signaling Technology), p-AKT (4060, Cell Signaling Technology), S6K (9202, Cell Signaling Technology), pS6K, (9204, Cell Signaling Technology), 4EBP1 (9644, Cell Signaling Technology) and p-4EBP1 (9451, Cell Signaling Technology) overnight at 4° C. After extensive washing, the membranes were incubated with the corresponding secondary antibody for 2 h at room temperature. The protein bands were visualized by using an enhanced chemiluminescence kit (Amersham Biosciences, Tokyo, Japan).

### Cell proliferation assay

Cell proliferation was assessed by the Cell Counting Kit-8 (CCK8) (Dojindo Laboratories, Kumamoto, Japan). In brief, cells were seeded in a 96-well plate at a density of 4×10^3^ cells/well and allowed to adhere. CCK-8 solution (10 μl) was added to each well, and the cells were cultured in 5% CO2 at 37° C for 2 h. Cell proliferation was determined by measuring the absorbance at 450 nm. Cell proliferation curves were plotted using the absorbance at each time point.

### Colony formation assay

Cells with a density of 500 per well were seeded in 6-well plates. Fresh culture medium was replaced every 3 days. After 2 weeks of incubation, individual colonies (> 50 cells/colony) were fixed and stained with 1% crystal violet for 1 h. Colonies were observed and counted, microscopically.

### Migration assays

A total of 4 × 10^4^ cells were plated in the upper compartment of Transwell^TM^ chambers (8 μm pore size, Corning, NY, USA) in serum-free medium. Fresh medium containing 10% FBS was added to the lower chamber. After incubation for 36 h, the cells in the lower membrane were fixed with paraformaldehyde solution for 15 minutes at room temperature and then stained with 0.1% crystal violet for 2 h. Three 20 × magnification fields were randomly chosen for counting the cell number under a microscope.

### Wound healing calculation

Caki-2 cells and A498 cells were plated in 6-well plates. A linear scratch wound was made by a 20-μl pipette tip in a confluent monolayer of cells. After 48 h of incubation in medium without FBS, the wound area was observed and photographed under a microscope.

### Cell cycle and apoptosis

Flow cytometry was utilized to profile the cell cycle and apoptosis. After trypsinization, ccRCC cells were harvested, washed, and fixed, and the cells were incubated in a solution with 10 mg/ml RNase and 1 mg/ml propidium iodide (KeyGen Biotech, Nanjing, China) at 37° C for 30 minutes in the dark. Finally, the cells were analyzed on a flow cytometer (BD Biosciences, USA). Cultured ccRCC cells were harvested and centrifuged, and then stained with an Annexin V-FITC and PI apoptosis detection kit (KeyGen Biotech, Nanjing, China) in the dark for 15 minutes at room temperature. Cellular apoptosis was assessed by a flow cytometer (BD Biosciences, USA).

### Tumorsphere formation

Caki-2 or A498 cells were seeded on ultralow attachment 6-well plates (Corning, NY, USA) at 5×10^3^ cells per well for primary tumorsphere formation. After incubation for 2 weeks, tumorspheres were collected and enzymatically dissociated by trypsin. For secondary tumorsphere formation, 2000 cells per well were plated in ultralow attachment 96-well plates again. Cells were grown in StemXVivo Serum-Free Media (R&D systems) supplemented with 2 U/ml heparin (H8060, Solarbio, Beijing) and 0.8 μg/ml hydrocortisone (G8450, Solarbio, Beijing). Two weeks later, tumorspheres were observed and analyzed under an inverted phase-contrast light microscope (Olympus).

### *In vivo* tumorigenicity assay

Xenograft mouse models using ccRCC cell lines have been well established by our team. Four- to six- week-old male BALB/c nude mice were obtained from Vital River Company (Beijing, China) for *in vivo* xenografts, and maintained under conditions as specified. Five mice were randomized into shCtrl, shNUPR1-1, and shNUPR1-2 groups and subcutaneously inoculated with Caki-2 and A498 cells. Tumor size was monitored by measuring the tumor volume every week with a caliper. The tumor volume was calculated as length × width^2^ × 0.52. Four weeks after inoculation, following euthanasia, the tumor was harvested, weighed, and imaged. All procedures were approved by the Animal Care and Use Committee at Shandong Provincial Hospital.

### Statistical analysis

SPSS 25.0 (IBM, Armonk, NY, USA) and GraphPad Prism 8.0 (GraphPad Software, San Diego, CA) were used for statistical analysis. All quantitative data are presented as the mean ± standard obtained from at least three independent experiments. Student’s *t*-tests or one-way ANOVA tests were used to evaluate the relationship between parametric variables. *Chi*-squared tests were used to assess the relationship between nonparametric variables. Significant prognostic predictors in univariate and multivariate analyses were performed using a Cox proportional hazards regression model. Survival probabilities were calculated by the Kaplan-Meier method and compared between groups using a log-rank test. In all analyses, *p* < 0.05 was considered statistically significant (*p < 0.05, **p < 0.01, ***p < 0.001).

### Ethic approval and informed consent

The study was approved by the Institutional Review Board of the Ethics Committee of Shandong Provincial Hospital (No. SWYX2020-256). The study protocol conformed to the ethical guidelines of the 1975 Declaration of Helsinki. The written informed consents from the patients were obtained from the patients to publish this manuscript. All protocols involving mice were approved by the Laboratory Animal Ethics Committee of Shandong Provincial Hospital (No. 2020-019).

### Consent for publication

All patients or their caregivers signed a consent form giving permission to use their anonymous data for research.

### Data availability

The datasets used and/or analyzed during the current study are available from the corresponding author on reasonable request.

## Supplementary Material

Supplementary Figures

Supplementary Table 1
