# Supplementary material for: NUPR1 is a novel potential biomarker and confers resistance to sorafenib in clear cell renal cell carcinoma by increasing stemness and targeting the PTEN/AKT/mTOR pathway
Source: Aging (Albany NY). 2021 May 24;13(10):14015–38. doi: 10.18632/aging.203012 (PMC8202846; doi:10.18632/aging.203012)
Supplement: Supplementary Figures [file aging-13-203012-s001.pdf]

## SUPPLEMENTARY FIGURES

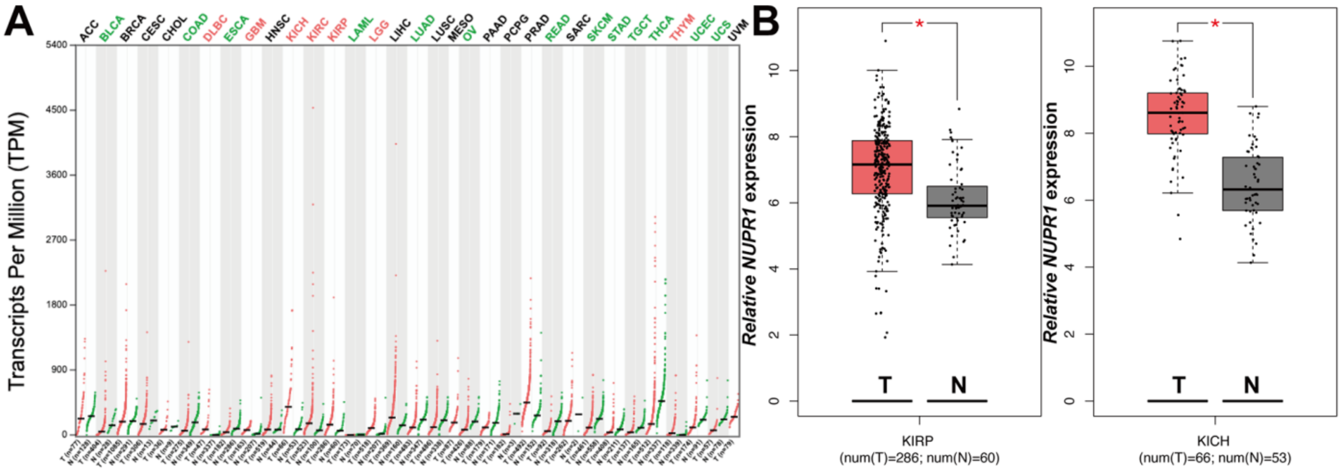


**Supplementary Figure 1. NUPR1 mRNA levels in malignancies.** (**A**) NUPR1 mRNA expression level in various cancer types in TCGA database. (**B**) Relative NUPR1 mRNA in TCGA-KIRP and KICH (http://gepia.cancer-pku.cn). (*$p$ < 0.05, **$p$ < 0.01, ***$p$ < 0.001). (N.S., No statistical significance). Kidney renal clear cell carcinoma; KICH: Kidney chromophobe cell carcinoma; KIRP: Kidney renal papillary cell carcinoma; TCGA: The Cancer Genome Atlas database.

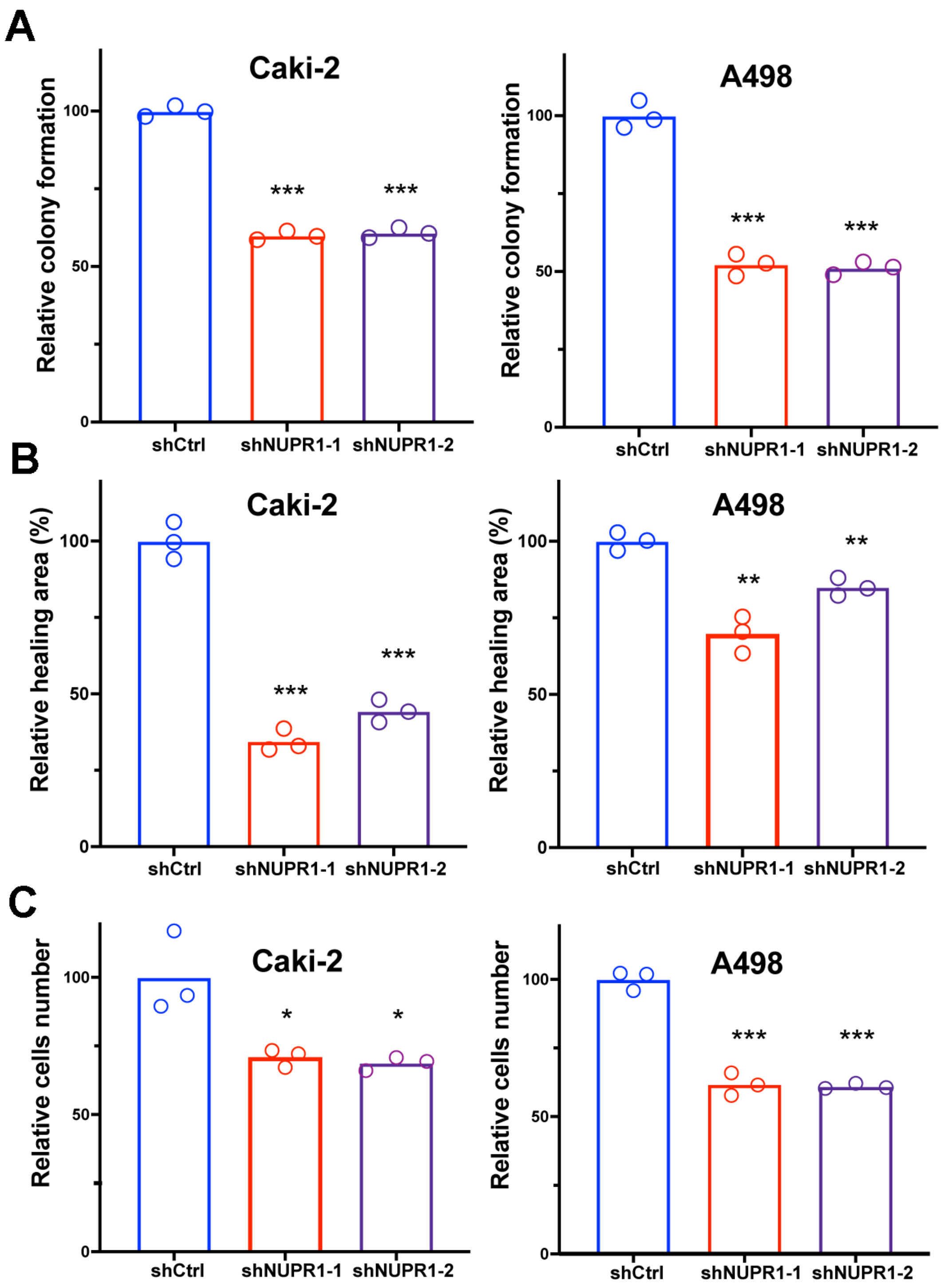


**Supplementary Figure 2. NUPR1 depletion suppressed the proliferation, migration and invasion in ccRCC.** (**A**) Histogram of colony formation assay for ccRCC cells with NUPR1 depletion. (**B**) Histogram the wound-healing assay of NUPR1 silencing on the migration of ccRCC cells. (**C**) Histogram of transwell experiment analysis of the effect of NUPR1 depletion on migratory and invasive abilities of ccRCC cells. (*$p < 0.05$, **$p < 0.01$, ***$p < 0.001$). ccRCC: clear cell renal cell carcinoma.

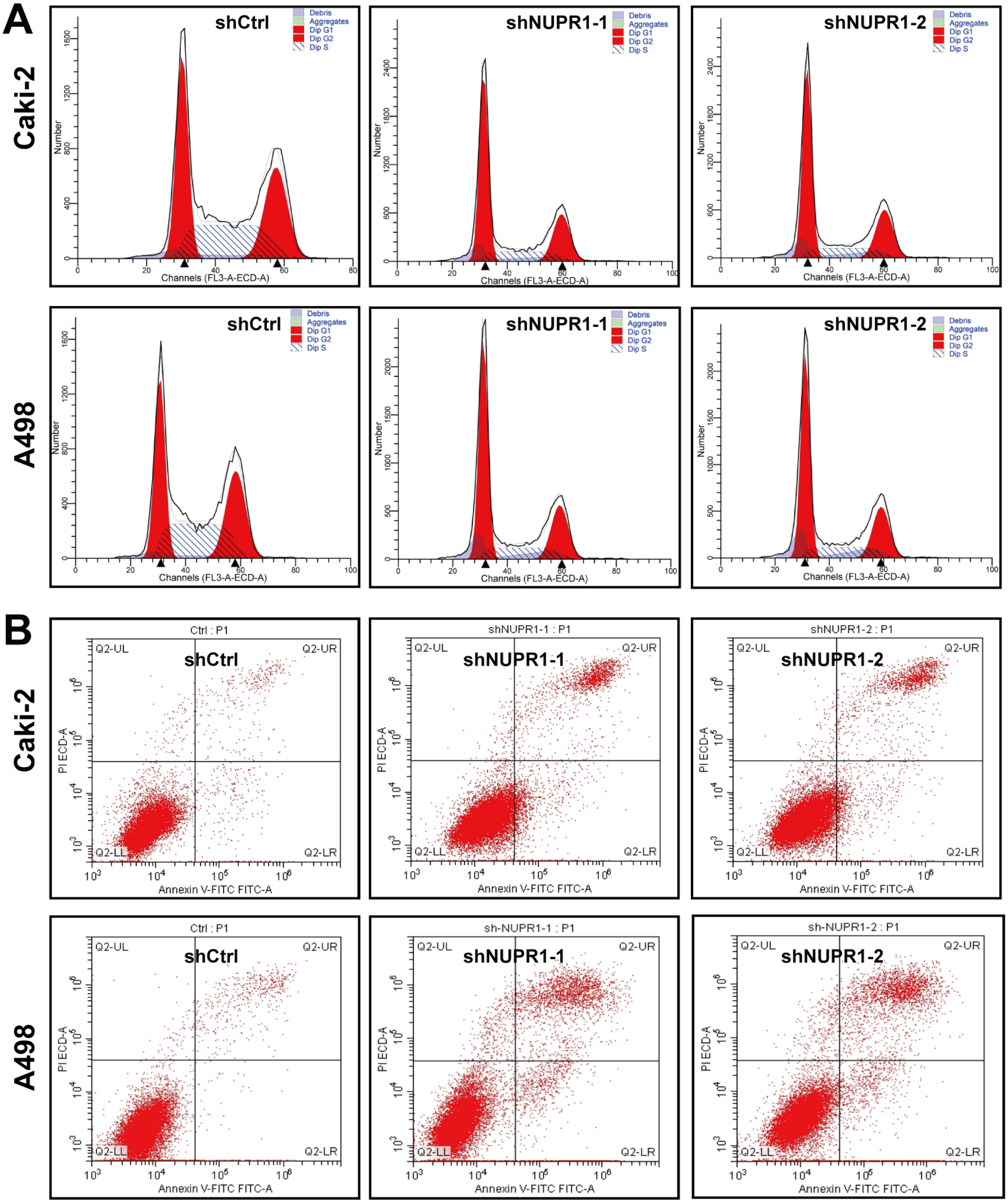


**Supplementary Figure 3. Effects of NUPR1 silencing on cell cycle and apoptosis.** (**A**) Effects of NUPR1 silencing on cell cycle regulation in ccRCC using flow cytometry. (**B**) Apoptosis assay of silencing NUPR1 in ccRCC by flow cytometry. (*$p < 0.05$, **$p < 0.01$, ***$p < 0.001$). ccRCC: clear cell renal cell carcinoma.

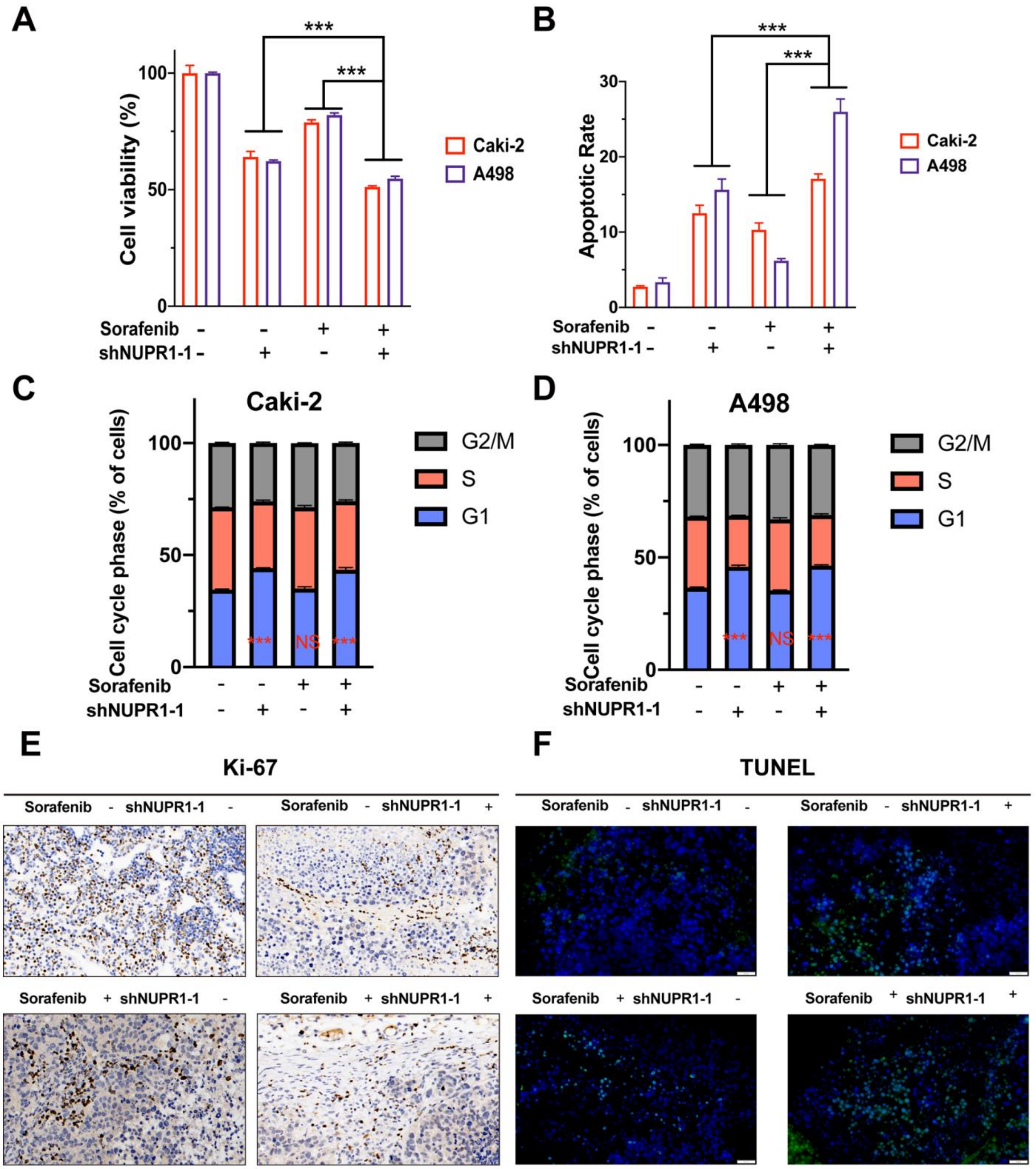


**Supplementary Figure 4. Effects of sorafenib and NUPR1 silencing on proliferation, cell cycle and apoptosis.** (**A**) The cells with NUPR1 silencing were treated with sorafenib (5μM) for 48 h. Cell viability was determined by CCK-8 assay. (**B**) Apoptosis assay of silencing NUPR1 with sorafenib treatment in ccRCC by flow cytometry. (**C**, **D**) Effects of NUPR1 silencing and sorafenib on cell cycle regulation in ccRCC using flow cytometry. (**E**) Ki-67 expression were detected by IHC in xenografts sections. (**F**) Apoptosis assessed by TUNEL in xenografts sections. (*$p < 0.05$, **$p < 0.01$, ***$p < 0.001$). ccRCC: clear cell renal cell carcinoma.

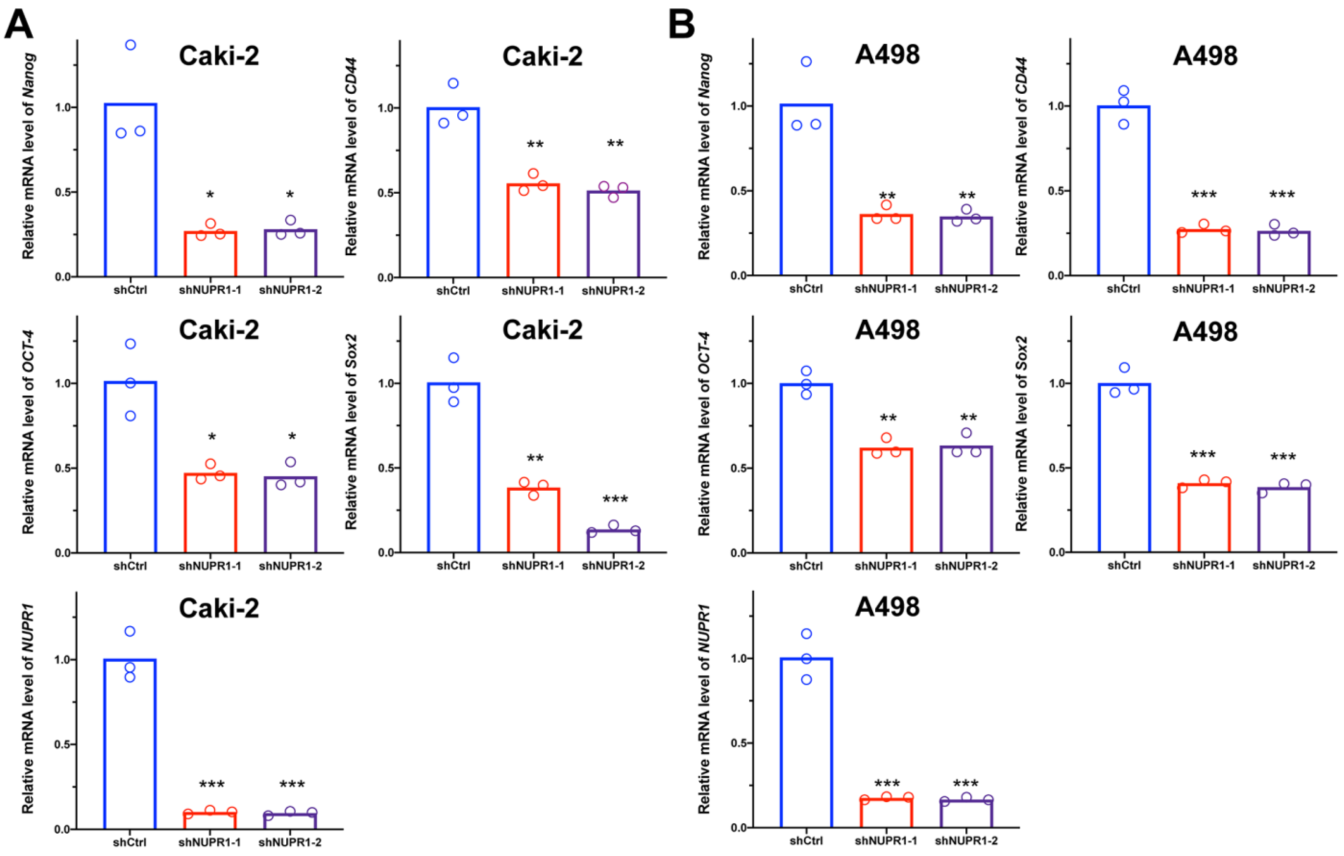


**Supplementary Figure 5. NUPR1 silencing mediated stem-related biomarkers mRNA transcription.** (**A**) NUPR1 silencing decreased stem-related biomarkers mRNA level in ccRCC cell Caki-2 verified by qRT-PCR. (**B**) NUPR1 silencing suppressed stem-related biomarkers mRNA level in ccRCC cell A498 analyzed by qRT-PCR. (*$p < 0.05$, **$p < 0.01$, ***$p < 0.001$). ccRCC: clear cell renal cell carcinoma; qRT-PCR: quantitative real-time reverse transcription PCR.
