# Supplementary material for: NUPR1 is a novel potential biomarker and confers resistance to sorafenib in clear cell renal cell carcinoma by increasing stemness and targeting the PTEN/AKT/mTOR pathway
Source: Aging (Albany NY). 2021 May 24;13(10):14015–38. doi: 10.18632/aging.203012 (PMC8202846; doi:10.18632/aging.203012)
Supplement: Supplementary Table 1 [file aging-13-203012-s002.pdf]

SUPPLEMENTARY TABLE

Supplementary Table 1. Primers sequences.

Sequence	Forward	Reverse
CD44	CAGCTCATACCAGCCATCCA	GCCTCATCTCCAGCTCTGTC
Nanog	CCCCTAATTTGTTGGTTGTGCT	GCTAATTTCCTTCTCCACCCCA
NUPR1	TCGGAGGTGGAGGCCG	GCCTCATCTCCAGCTCTGTC
OCT4	CCTTCGCAAGCCCTCATTTC	TAGCCAGGTCCGAGGATCAA
SOX2	CATGAAGGAGCACCCGGATT	ATGTGCGCGTAACTGTCCAT
